# The GOES Time Code Service, 1974–2004: A Retrospective

**DOI:** 10.6028/jres.110.008

**Published:** 2005-04-01

**Authors:** Michael A. Lombardi, D. Wayne Hanson

**Affiliations:** National Institute of Standards and Technology, Boulder, CO 80305

**Keywords:** broadcasting, Coordinated Universal Time (UTC), orbit prediction, satellites, timekeeping

## Abstract

NIST ended its Geostationary Operational Environmental Satellites (GOES) time code service at 0 hours, 0 minutes Coordinated Universal Time (UTC) on January 1, 2005. To commemorate the end of this historically significant service, this article provides a retrospective look at the GOES service and the important role it played in the history of satellite timekeeping.

## 1. Introduction

After nearly 30 years of continuous operation, NIST ended its Geostationary Operational Environmental Satellites (GOES) time code service at 0 hours, 0 minutes Coordinated Universal Time (UTC) on January 1, 2005. This event marked the end of an important chapter in the history of timekeeping. The GOES time code service is historically significant for at least two reasons: it was the first time code service ever broadcast via satellite, and was the first time code service of any type that provided transmitter position data in addition to the time. The position data made it possible for receivers whose position was also known to compute and remove the signal path delay and improve the timing accuracy.

NIST began preparing for the end of the GOES time code service in the mid-1990s, when it was clear that Global Positioning System (GPS) satellite timing receivers provided better accuracy and reliability than GOES at a lower cost. Nearly all GOES receivers were being replaced by GPS units, making the decision to eventually stop the service an easy one. However, during their heyday, GOES time code receivers were widely used by the electric power and aviation industries; and it is estimated that more than 10 000 receivers were sold, produced by at least three different manufacturers. This article provides a retrospective view of the GOES time code service, beginning with a look at the early days of radio time code broadcasts and the first satellite timing experiments.

## 2. Ground Based Time Code Broadcasts

Wireless time signals existed long before satellites; in fact, telegraphic time signals from ground based transmitters were broadcast beginning in 1903 by the United States Navy [1, 2]. NIST [then the National Bureau of Standards (NBS)] has participated in this arena for many years, beginning with standard frequency broadcasts from radio station WWV in 1923 [3], and later adding telegraphic [4], voice [5], and digital time codes [6] to its broadcasts. From the beginning, it was known that wireless time signals are delayed as they travel the path from the transmitter to the receiver, and that the accuracy of the received time signal can be no better than the knowledge of the path delay. Signals originating from a ground based transmitter have path delays that are difficult to estimate, since the delay continually changes due to changing ionospheric conditions. Some of these problems are reduced by signals that do not reflect off the ionosphere, such as line-of-sight signals with small coverage areas, and ground-wave signals in the low frequency (LF) part of the radio spectrum below 300 kHz. However, it was clear even in the pre-satellite days that a time signal broadcast from the sky high above the Earth, where there was a clear, unobstructed path between the transmitter and receiver, would potentially be more accurate than any ground based signal.

## 3. The First Satellites and Early Satellite Timing Experiments

The Space Age began with the launch of the Russian satellite *Sputnik 1* in October 1957, followed by the launch of the first American satellite, named *Explorer 1*, just four months later. The earliest satellites were used for solar and atmospheric studies, but the emphasis quickly turned to telecommunications. The U. S. Army’s *SCORE* (Signal Communication by Orbiting Relay Equipment) was perhaps the first telecommunications satellite, broadcasting prerecorded Christmas wishes from President Eisenhower after its launch in December 1958. However, *Echo 1*, a 30.5 m diameter mylar balloon launched by the National Aeronautics and Space Administration (NASA) in August 1960 is generally credited with ushering in the age of satellite telecommunications [7]. A passive-relay satellite, *Echo 1* simply served as a “mirror” that reflected radio signals back to Earth, albeit with an approximately 180 dB loss in signal strength [8]. *Echo 1* enabled the first satellite telephone link in February 1962, and in April of that same year enabled the broadcast of a television program from California to Massachusetts [9].

Early propagation studies conducted using *Echo 1* in 1960 [10] are sometimes identified as the first satellite timing experiments, but the first precise time experiment was probably performed via the active-relay satellite *Telstar 1* in August 1962, roughly one month after that satellite was launched. This experiment allowed the United States Naval Observatory (USNO), and the United Kingdom’s National Physical Laboratory (NPL) and Royal Greenwich Observatory (RGO) to perform a transatlantic clock comparison using a new technique called two-way satellite time transfer. The ground stations used for the time transfer experiment were located in Andover, Maine and Goonhilly Downs in the United Kingdom. The results were impressive. Uncertainties of just a few microseconds were reported, about 1000 times smaller than the uncertainties previously reported for transatlantic clock comparisons using ground based radio signals, which had been limited to about 2 ms [11].

## 4. Geostationary Satellites

The *Telstar 1* experiments represented a true breakthrough in time transfer accuracy, but they didn’t allow a continuous timing signal to be broadcast. Since the satellite was in a relatively low Earth orbit of roughly 900 km × 5700 km, its orbital period was about 158 min. Thus, its signals could be received only during the short periods of time when the satellite was “visible” to the receiver. The longest time transfer passes reported in [11] were less than 1 hour in duration. Creating a satellite time service would require satellite signals that were always available. This meant that either a geostationary satellite or an orbiting constellation of satellites all broadcasting the same time (such as today’s GPS) would be needed.

The concept of a geostationary or synchronous orbit was perhaps first introduced by the celebrated science fiction writer Arthur C. Clarke, in a 1945 letter to the editors of *Wireless World* [12]:

An “artificial satellite” at the correct distance from the earth would make one revolution every 24 hours; i.e., it would remain stationary above the same spot and would be within optical range of nearly half the earth’s surface. Three repeater stations, 120 degrees apart in the correct orbit, could give television and microwave coverage to the entire planet.

Later that year, Clarke published an article in *Wireless World* [13] that described the basic principles of geostationary orbit with a surprising amount of scientific detail. As we know today, a geostationary orbit is a circular orbit in the Earth’s equatorial plane. Any point in the orbit will revolve about the Earth in the same direction and with the same period as the Earth’s rotation. An object in geostationary orbit will remain directly above a fixed point on the equator at a distance of approximately 42 164 km from the center of the Earth, or approximately 35 786 km above mean sea level.

The first successful geostationary satellite, *Syncom 3*, was launched about 20 years after Clarke’s writings (in August 1964), fulfilling his prediction for television coverage. Among other things, *Syncom 3* was used to transmit television signals from the 1964 Olympics in Tokyo, Japan to the United States [7].

## 5. The Beginning of the NBS Satellite Program

NBS had begun work related to satellite time transfer as early as 1963 by measuring radio transmissions from the United States Navy’s *Transit 4A* navigation satellite [14], and began time synchronization experiments using geostationary satellites in the mid 1960s. Time synchronization experiments using VHF (135.6 MHz downlink) transmissions from the NASA *Application Technology Satellite (ATS-1)* were conducted in 1967. These measurements compared clocks located in Colorado, Hawaii, and California by making round trip time delay measurements via the satellite transponder, resulting in time synchronization uncertainties of just a few microseconds over a 10 d period [15].

By 1968, attention at NBS had turned at least partially to one-way time transfer. The time transfer experiments previously described in [11] and [15] involved two-way transmissions between the local and remote clocks, relayed by the satellite transponder. The two-way technique [16] is still widely used for comparing high accuracy clocks used as national time standards, but requires each participating ground station to be able to transmit signals through the satellite. In a one-way time transfer system, the satellite broadcasts time that can be used to synchronize any number of clocks located in the coverage area. Just as anyone with a radio could listen to a local station, anyone with an appropriate receiver could use the time signal to synchronize a local clock. One-way broadcasts would allow NBS to create a continuously running time service usable by the general public, similar to the services already provided by its ground based stations WWV, WWVB, and WWVH, but with substantially better accuracy.

One-way time transfer experiments conducted using *ATS-1* in 1968 revealed that the key problem was predicting the radio propagation delay from the reference clock on Earth through the satellite (uplink), and from the satellite back to a receiver on Earth (downlink). Many factors contributed to the uncertainty of this delay measurement, including delay variations in satellite and ground station equipment, and in ionospheric conditions. However, the largest contribution to uncertainty was the knowledge of the satellite’s position. Using a NASA computer program that computed the position of *ATS-1* from a set of Keplerian orbital elements (described in Sec. 7), it was found that the delay could be estimated to within 60 µs using a set of orbital elements that were a week old, and within 10 µs if the orbital elements were generated by NASA immediately prior to the experiment [17]. This was many times better than the uncertainty of delay measurements from stations such as WWV, where propagation delays were often difficult to estimate to better than 1 ms.

These results were quite promising, and by 1970, NBS researchers had announced their intentions to create a satellite time code service that was “relatively inexpensive, simple-to-operate, and easily understood”. It was anticipated that a service would be available in a “few” years. Unlike future satellite systems such as GPS, where atomic oscillators would be carried on-board the satellite, the master clock would be on the ground. The satellite itself would not carry a clock or oscillator; it would simply relay the time signals it received from the ground station [18]. The reference for the service would be UTC as kept at NBS, or UTC(NBS).

**Fig. 1:**
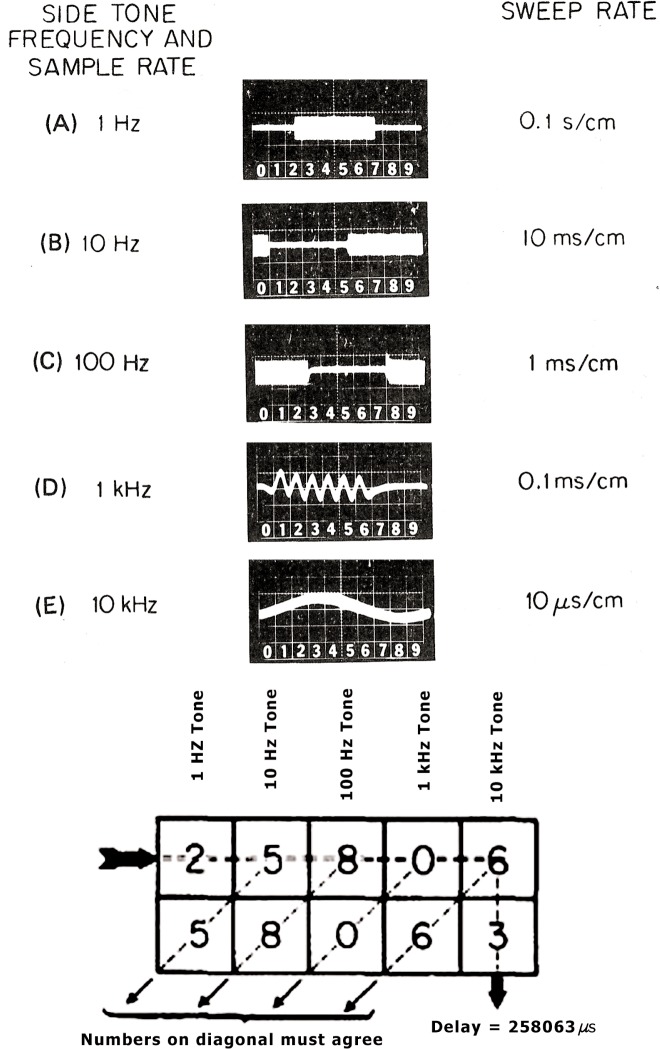
Side-tone satellite ranging.

By 1971, NBS had conducted timing experiments using several different geostationary satellites including the NASA ATS satellites, the Lincoln Experimental Satellite-6 (*LES-6*), and the military Tactical Communications Satellite (*TACSAT*). During some of these experiments, a technique known in the satellite tracking field as side-tone ranging was used to measure propagation delay. The master clock station transmitted a series of sine wave tone “bursts” obtained by dividing a 1 MHz cesium frequency standard. These tone bursts were sent at rates of 1 Hz, 10 Hz, 100 Hz, 1000 Hz, and finally a continuous tone of 10 KHz. When the receiving station received these tones, it measured their phase shift relative to tones derived in the same fashion from their local clock. The values were then combined to compute the total delay. The lowest frequency audio tone resolved the ambiguity of delay while the highest frequency tone enabled resolution down to 1 µs (Fig. 1). The measured delays agreed fairly well, typically to within tens of microseconds, to theoretical delay estimates made using the coordinates of the transmitter, satellite, and receiver [19, 20].

## 6. WWVS

The first attempt at creating a NBS satellite service involved rebroadcasting audio signals from WWV via NASA’s geostationary *ATS-3* satellite, a project that was known as WWVS. The uplink to the satellite was sent at 149.245 MHz from the NBS laboratories in Boulder, Colorado, and sent back to Earth on a 135.625 MHz downlink. Broadcasts took place between 1700 and 1715 UTC, and 2330 to 2345 UTC every Monday through Friday (excluding holidays) beginning on August 1, 1971. *ATS-3* was located at 70° west longitude, and its signal covered 40 % of the Earth’s surface, including North and South America, major parts of the Pacific and Atlantic oceans, and portions of Europe and Africa. The voice announcements were delayed by about 250 ms. However, by working with NASA’s orbital elements for *ATS-3*, NBS was now routinely predicting the delay from Boulder to any point in the satellite’s coverage area to within 10 µs to 20 µs [21,22].

**Fig. 2:**
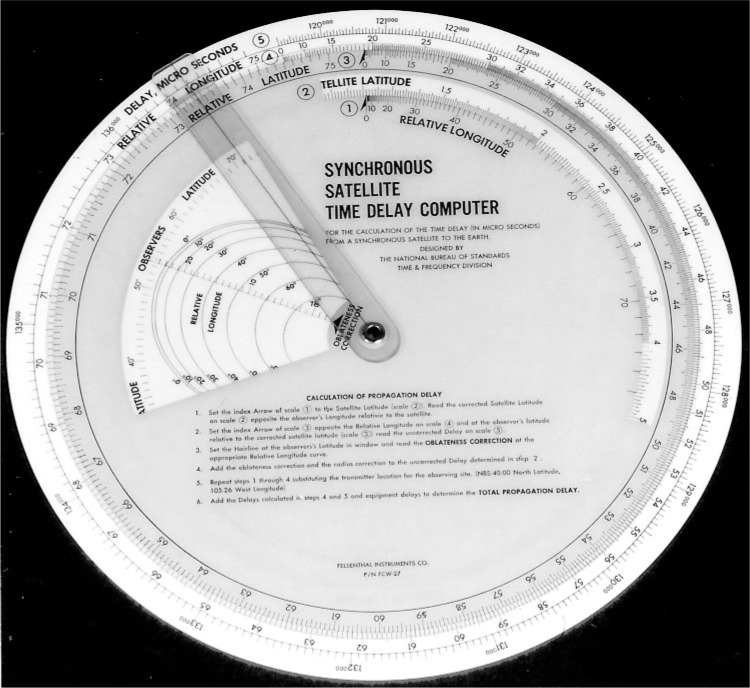
Synchronous satellite time delay “computer”, 1973.

For customers who required high accuracy timekeeping, NBS designed a special purpose delay “computer” in the form of a circular slide rule [23]. This slide rule could be printed on paper, cut out, and assembled; and was later printed on laminated plastic and distributed free of charge to those who requested it (Fig. 2). Beginning in 1972, the voice announcements included the satellite’s longitude and latitude and a radius correction. Using this information, along with the longitude and latitude of their receiver, the customer could manipulate the slide rule and obtain a path delay estimate. Delays estimated with the slide rule had a standard deviation of < 25 µs when compared to actual delays measured from Boulder over a period of several months, and the combined uncertainty of the signal was < 50 µs. This was confirmed at monitoring stations installed in Colorado, Massachusetts, Peru, and Brazil, where the WWVS broadcast was received and compared to UTC synchronized clocks [24, 25].

Although it represented an important step forward in satellite timekeeping, WWVS never became an NBS service. The experiment was discontinued in August 1973, about 2 years after it began, but plans to develop a service continued. WWVS was placed into NBS budget requests for several years, was granted a frequency allocation of 400.1 MHz from the International Telecommunications Union (ITU), and NBS and NASA developed a Memorandum-of-Agreement (MOA) to provide the service. However, the possibility of WWVS formally ended in 1977 when the Director of NBS terminated the MOA, indicating that the “satellite service would place a significant additional financial burden on NBS ….”, and that the “satellite service would depend on the continued availability of suitable satellites which are primarily dedicated to other services [26]”. By this time, the GOES satellite service had already been launched.

## 7. The GOES Service Begins

While the WWVS experiments were still in progress, NBS had begun work on another type of one-way satellite broadcast service, publishing a feasibility study in 1973 [27]. This proposed service would use the Synchronous Meteorological Satellites (SMS), the first of which was to be launched by NASA in May 1974. Once these satellites were functioning properly they were to be turned over to the National Oceanic and Atmospheric Administration (NOAA), and future satellites launched by the program would be renamed GOES (Geostationary Operational Environmental Satellites).

**Fig. 3:**
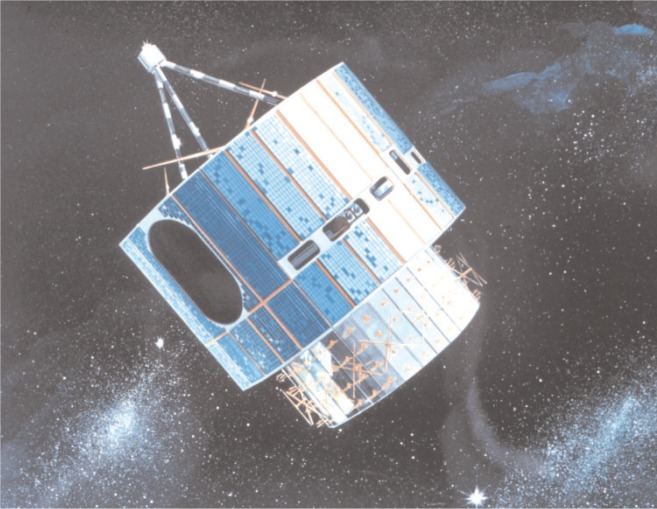
GOES satellite, 1975.

**Fig. 4:**
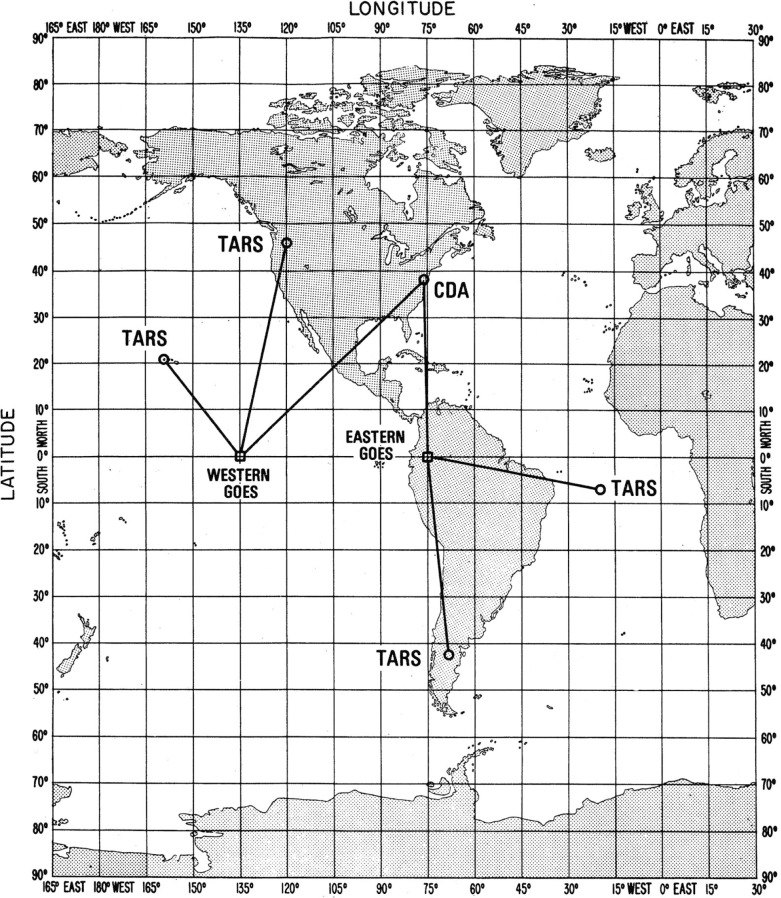
Satellite ranging network using trilateration.

The SMS/GOES satellites (Fig. 3) were designed to collect information about the weather. Their objective was to sense meteorological conditions from a fixed location above the Earth and to deliver this data to operational forecasters and private interests on the ground. Part of the GOES mission was to take pictures of storm patterns, frontal systems and the like. Since it was necessary to accurately locate these pictures with respect to Earth longitude and latitude, the position of the satellite had to be precisely known. To accomplish this, a ranging system was developed for NOAA under a NASA contract. The ranging system had a theoretical precision of about 1 m, and an accuracy limited mainly by the real time knowledge of the effects of the ionosphere and the troposphere. This ranging system was based on a concept termed trilateration. It worked by making ranging, or time delay measurements from the Earth to the satellite from three widely separated ground stations. The primary tracking station, called the Control and Data Acquisition Station or CDA, was located at the NOAA facility at Wallops Island, Virginia, near the city of Chincoteague (Fig. 4).

Two GOES satellites were continuously tracked. The western satellite, or GOES-West, was located at 135° west longitude. It was tracked from the CDA and sites in the states of Washington and Hawaii. The eastern satellite, or GOES-East, was located at 75° west longitude. It was tracked from the CDA, and stations in Santiago, Chile and Ascension Island in the South Atlantic. The ground stations other than the CDA were unmanned and known as Turn-Around Ranging Stations (TARS) [28]. This system of three ground stations provided three slant ranges to the satellite that allowed the location of the satellite to be determined geometrically (Fig. 4).

Data collected through trilateration was used to generate the six Keplerian orbital elements (*a*, *e*, *i*, Ω, *ω*, *M*) that described the shape and orientation of the satellite’s orbit at a given epoch. The semi-major axis, *a*, is the average distance in kilometers from the center of the mass of the Earth to the satellite. For the geostationary GOES satellites, *a* is nominally 42164 km. The eccentricity, *e*, is a measure of how close the orbit is to being circular. A perfect circle would have an eccentricity of 0. The orbit inclination angle, *i*, is the angle between the orbital plane of the satellite and the equatorial plane of the Earth, and is always near 0° for GOES. The right ascension of the ascending node, *Ω*, is the angle from the vernal equinox to the ascending node of the satellite. The ascending node is the place where the northbound satellite crosses the equator. The argument of perigee, *ω*, is the angle from the ascending node to the perigee, or the point on the orbit nearest to Earth. The mean anomaly, *M*, is an angle describing where on the ellipse the satellite is at the epoch. At perigee, the mean anomaly is 0°. At the highest point of the orbit (apogee), it equals 180°. Once these six orbital elements were obtained, it was straightforward to calculate the satellite’s position at a given epoch, and to estimate the position (with increasing uncertainty) for a number of days after the epoch. This allowed the position of GOES-East and GOES-West to be precisely known, making the satellites a logical platform for a time code service.

**Fig. 5:**
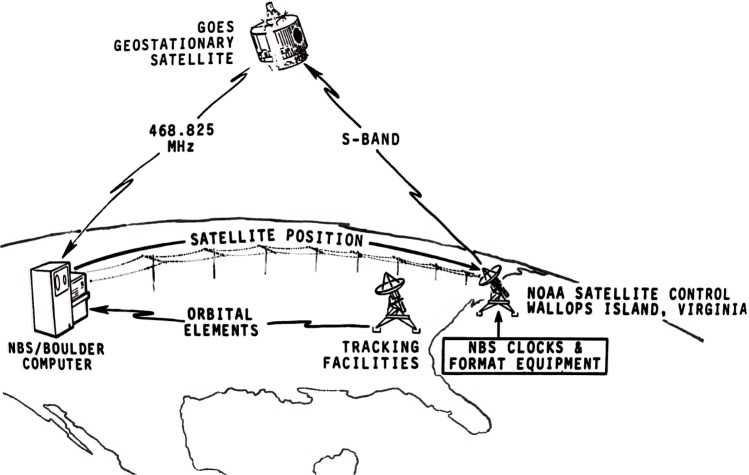
GOES time code distribution system.

The GOES system also offered a convenient way to send time information to and from the satellites (Fig. 5). In addition to their continuous photography of the Earth’s surface and the collection of space data related to Earth/Sun interaction, the GOES satellites also collected data from remote sensors located on Earth. These remote sensors, called data collection platforms (DCPs), are used to monitor flood, rain, snow, tsunami, earthquake, and air/water pollution conditions [28]. Some DCPs are equipped with both a receiver and a transmitter. When an interrogation message is received from the satellite, they transmit their stored data using frequencies near 401 MHz through the satellites and back to the CDA at Wallops Island. The CDA continuously relays interrogation messages to both GOES-East and GOES-West via two 18.3 m diameter parabolic antennas at an S-band frequency of approximately 2034 MHz. The interrogation messages are sent back to Earth at downlink frequencies of 468.825 MHz from GOES-West and 468.8375 MHz from GOES-East at a data rate of 100 bits per second, using just 400 Hz of bandwidth [29, 30].

NOAA agreed to provide space in the interrogation message for a NBS time code. This decision was beneficial to both agencies. NOAA realized that the collected data would be more valuable if it were time stamped, and NBS realized that GOES provided the medium it needed to launch its satellite time code service. NBS time was broadcast from SMS-1 immediately after its launch in May 1974, continuously broadcast from the GOES satellites on an experimental basis beginning in 1975 [28], and declared an operational service by NBS and NOAA in May 1977 [29].

**Fig. 6:**
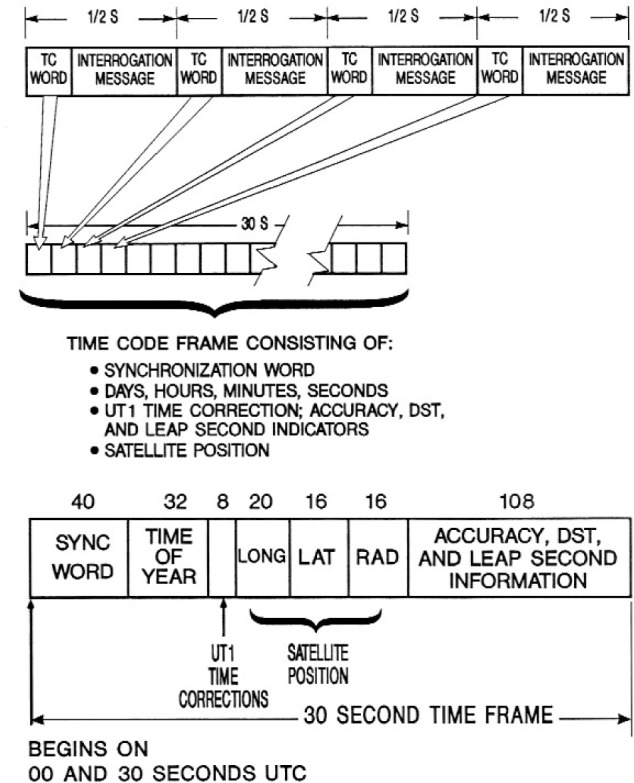
GOES interrogation message and digital time code formats.

Each interrogation message consists of 50 bits of data, requiring 0.5 s to send. The data are Manchester encoded and then modulated on to the carrier using ±60° coherent phase shift keying (CPSK) [28, 29]. The first 4 bits of the interrogation message consists of a binary coded decimal (BCD) time code word. This was followed by a 15 bit maximum length sequence (MLS) used for message synchronization, and a 31 bit address for a particular DCP. When a DCP receives and recognizes its unique address it sends its data to the satellite. The time code frame designed by NBS consisted of 60 time code words (240 bits), and required 30 s to transmit. The time code frame contained a 40 bit synchronization message, a 32 bit time-of-year message including the day of year, and the UTC hour and minute; an 8 bit message containing the current offset between UTC and the astronomical time scale UT1, and a 52 bit ephemeris message containing the satellite’s latitude, longitude, and height above the Earth’s surface minus a bias of 119 300 µs [31]. The remaining 108 bits were originally unused, but by 1984 were used to store the year, expected-accuracy indicators, system status information, and indicators for daylight saving time and leap seconds [32] (Fig. 6).

NBS time was supplied to the satellites by time code generators (TCGs) designed at NBS and located at the CDA. Three TCGs were installed for redundancy. All continuously kept time, but only one was on the air. A system was designed that tripped an alarm if any one of the three TCGs disagreed with the other two by more than 20 µs. If the on-air TCG was in error, CDA personnel switched to one of the other units. The TCGs accepted a 5 MHz time base signal from a cesium oscillator, which was divided down to produce the 100 Hz data clock frequency. The TCGs also accepted a 1 pulse per second (pps) signal that was synchronized to agree with UTC(NBS) and later UTC(NIST), as discussed in Sec. 9. The TCGs were controlled by telephone, using 300 baud modems (designed by NBS in the pre-PC days) from the NBS laboratories in Boulder, Colorado. Satellite position data were uploaded over this link from Boulder (Sec. 10). The TCGs stored 240 h of position data, but included firmware to interpolate between the hourly position estimates so that the satellite position could be updated more often. The original TCG configuration updated the position data to the satellites once every 30 min [28]. This was later reduced to 4 min [33], and eventually to 1 min [32]. The more frequent position updates smoothed the received data and improved the quality of the service.

The GOES time code service was designed to operate in two modes, known as uncorrected mode and corrected mode. In uncorrected mode, the satellite ephemeris included in the time code was ignored by the receiver. It was known that the average free space propagation delay from the CDA through the satellite to any given point in the coverage area would be near 260 ms. Therefore, the TCGs sent the time out 260 ms early, reducing the maximum possible time error anywhere in the coverage area to ±16 ms [34]. For example, the delay from the CDA to Boulder through GOES-East was near 252 ms, resulting in the uncorrected time arriving about 8 ms early; through GOES-West the delay was near 261.5 ms, resulting in the uncorrected time arriving about 1.5 ms late. Corrected mode required the receiver to have a microprocessor so it could read the ephemeris data code, and compute the free space delay from the satellite to the receiver’s antenna (the receiver’s coordinates were entered by the user and stored in memory). This improved the timing uncertainty to ±100 µs [30], and allowed GOES to become the first time code service of any type that allowed receivers (Sec. 8) to automatically correct for path delay.

**Fig. 7:**
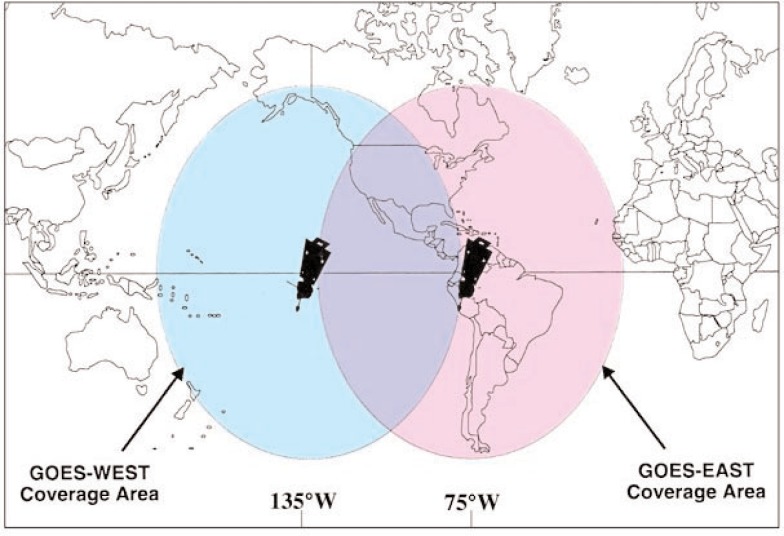
The GOES time code service coverage area.

The coverage area for the GOES time service was larger than other time code services in the pre-GPS era (Fig. 7). It included nearly all of North and South America, and most of the United States received coverage from both satellites. Unlike reception of the ground based NBS time stations, GOES reception was equally good during the day or night, and since the receiving antennas were wide beam, pointing the antenna was not that critical. Receivers in the United States could usually automatically switch to the East satellite if West was not available, and vice versa, without moving the antenna.

## 8. The NBS GOES Receiver Leads to Commercial GOES Receivers

Once the time code service had begun, it was obvious that GOES time code receivers must be made available before the new service would acquire any customers. To promote the service, NBS designed a receiver intended to stimulate the private sector into producing their own. The NBS unit could be built for less than $200 in parts, and represented a significant technical achievement in the days prior to the advent of the personal computer. It used the 4 bit Intel 4004^1^ as its central processing unit, now generally regarded as the first microprocessor ever developed. The extremely efficient assembly language firmware resided in just 512 bytes of read only memory (ROM), and the random access memory (RAM) occupied just 40 bytes! The microprocessor clock ran at 4.096 MHz, which was frequency divided by 40960 (8 × 2 × 16 × 16 × 10) to produce 100 Hz and then phase locked to the received data clock from the satellite. The phase locked 100 Hz served as the time base for the microprocessor time-of-year (TOY) clock.

**Fig. 8:**
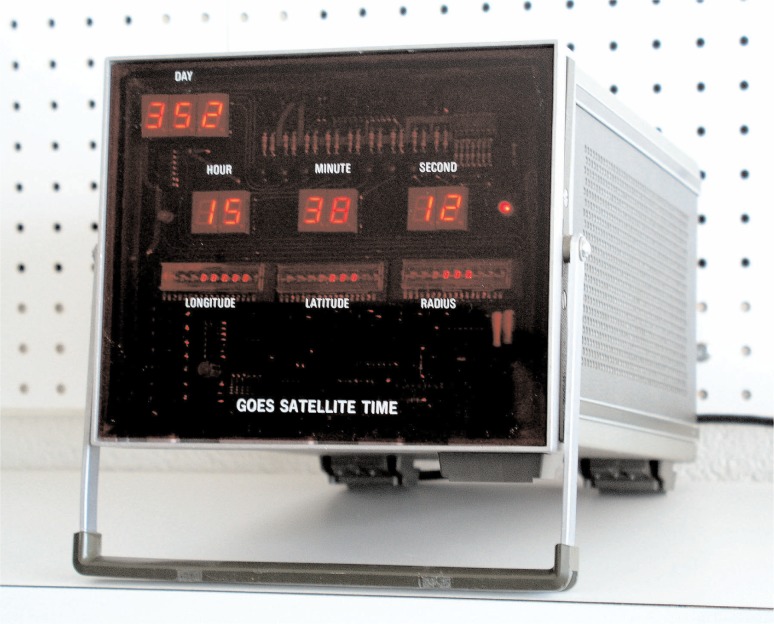
NBS satellite controlled digital clock, 1976.

**Fig. 9:**
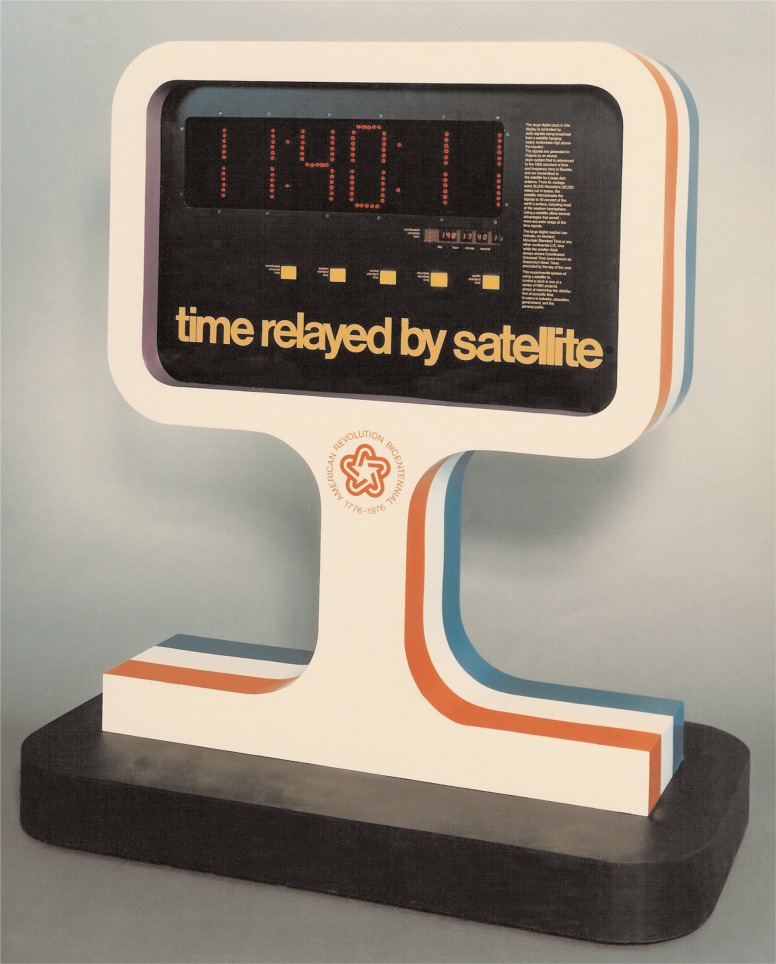
GOES controlled bicentennial time display, 1976.

Complete plans for a GOES time code receiver, including schematics and assembly language source code, were published in *NBS Technical Note 681* [35] and widely distributed. As a result of this work, a United States patent was awarded to NBS engineers Cateora, Davis, and Hanson in 1977 for their *Satellite Controlled Digital Clock System* [36]. Figure 8 shows the original satellite clock receiver built by NBS around 1976. Figure 9 shows a GOES controlled time display built for the United States bicentennial celebration in 1976.

The original receiver operated in uncorrected mode. *NBS Technical Note 1003* [37], published in 1978, contained the plans for a GOES “smart clock” that operated in corrected mode, making automatic path delay corrections. This clock added a second Intel 4004 microprocessor and a math chip removed from an early scientific calculator to the previous decoder clock shown in Fig. 8 [35]. The new hardware was used for the calculation of the free-space propagation delay from the CDA to the clock via the satellite. This delay value was used with a delay generator to compensate for the free-space path delay.

**Fig. 10:**
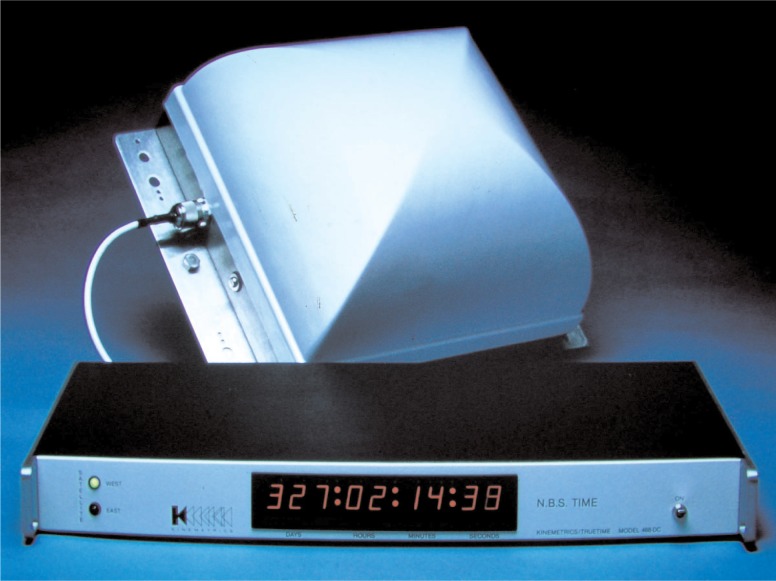
A commercial GOES time code receiver and antenna.

The published NBS receiver designs quickly generated interest in the commercial sector. It was noted in 1978 that NBS had been contacted by “more than 18 manufacturers” who were interested in building GOES time code receivers and by then, several commercial models were already available [38]. At least three manufacturers, including Arbiter Systems, TRAK Microwave, and True Time, made long term commitments to manufacture and sell GOES time code receivers; and it is estimated that over 10 000 commercial receivers were sold. The cost of a GOES time code receiver and antenna generally ranged from about $2000 to about $7500 U. S. dollars, depending upon the number of features included. Arbiter and True Time produced several different models and continued to sell and support GOES receiver and antenna products well into the 1990s (Fig. 10).

## 9. Time and Frequency Control and Uncertainty of the GOES Time Code Service

The frequency and time reference for the GOES service was usually a cesium oscillator, but rubidium oscillators were sometimes used. These oscillators were originally owned and maintained by NIST, but in the later years they were provided by NOAA. The TCGs were referenced to the atomic oscillator frequency and set to be 260 000 µs ahead of UTC(NIST), with an uncertainty of ±10 µs throughout the lifetime of the service [32], and ±1 µs during the last nine years (1996–2004). The TCGs were adjusted from Boulder in time increments of 0.2 µs, the period of the 5 MHz time base.

GOES time at the CDA was synchronized by NIST personnel using a number of techniques throughout the years, including the use of television signals from nearby Norfolk, Virginia, LORAN-C transmissions from Cape Fear, North Carolina [29, 30], portable clock carries from Boulder to Wallops Island [39], and eventually through the use of GPS signals [32]. The performance of the station clock was continuously monitored from Boulder using a remote controlled data logger [40], which was replaced by more modern equipment in 1999.

**Fig. 11:**
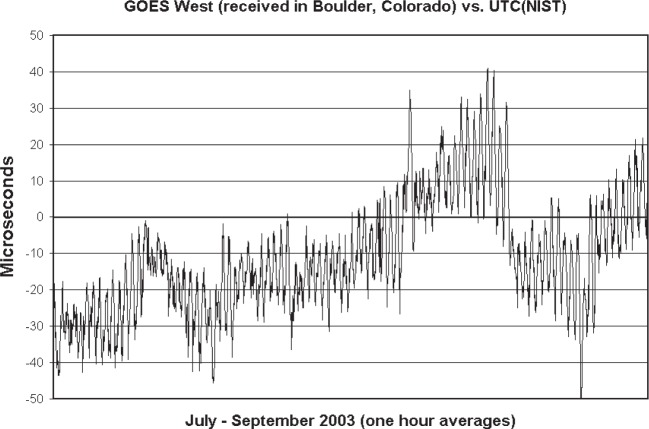
GOES-West as received in Boulder and compared to UTC(NIST).

The quality and age of the orbital elements always limited the timing uncertainty of the GOES service more than the performance of the station clock. In the 1980s, budget cuts forced NOAA to drop the trilateration ranging network and to make position estimates using images of the Earth transmitted by the satellites every 20 min. This resulted in poorer quality orbital elements [41]. Other incidents that increased the timing uncertainty were satellite maneuvers, where NOAA sometimes moved the satellites to a new position before new orbital elements were available and the position data in the TCGs could be updated; and solar eclipse periods, when the time code was sometimes moved to a spare satellite for periods as long as two hours each day [32]. Despite these challenges, the stated ±100 µs time uncertainty was usually met throughout the lifetime of the service, from the early days [33, 39] until the end. Figure 11 illustrates this by showing the results of a 3 month comparison between GOES-West as received in Boulder and UTC(NIST) in 2003. The noise visible in the received data is not random, and was mostly caused by errors in the delay computations due to imperfections in the predicted satellite orbit as discussed in the next section.

## 10. Orbit Prediction Software

Orbit prediction software was always an essential element of the GOES time code service. The GOES satellite orbits were not stationary but changed with time as they were perturbed by several effects, the major ones being solar and lunar gravitational attractions, solar radiation pressure, and the inhomogeneous gravitational field of the earth. These perturbations can be modeled reasonably well, but the accuracy of the orbit predictions deteriorates with time due to residual imperfections in the modeling. High quality orbit prediction software that reduced these residual imperfections to a minimum was necessary for the service to meet its ±100 µs timing specification, particularly because it was sometimes necessary to use orbital elements that were more than one month old.

The orbit prediction program originally used by the GOES service was a modified version of NASA’s Goddard Trajectory Determination System (GTDS). This software consisted of about 40 000 lines of Fortran code [41], and was installed on a large Control Data Corporation 6600 mainframe computer in Boulder [29]. It was later installed on a similar computer at NOAA’s facility in Suitland, Maryland, and if the Boulder computer was unavailable, the Suitland computer was accessed from Boulder. The input data for the software consisted of the six Keplerian orbital elements and their epoch [32]. The position estimates for the 240 h following the epoch were originally output on punched cards and brought to another computer where the cards were read into a file and then uploaded to the CDA, a slow process that new technology soon eliminated. The orbital elements were sent by NOAA via teletype until the mid-1990s, and in later years by email.

In the early 1990s, it was announced that both of the mainframe computers that ran *GTDS* were to be shut down, and NIST had to find new orbital prediction software. As part of a cooperative research program with the National Physical Laboratory in India (NPLI), NIST was given source code for the orbital prediction program used by NPLI’s Indian National Satellite (INSAT) time service. Unlike *GTDS*, which was capable of predicting all types of orbits, the NPLI software was streamlined to work with geostationary orbits only. This source code was modified for the GOES data format and a personal computer software application called *GOESTRAK* was created, replacing *GTDS* in late 1992. *GOESTRAK* used a much smaller, more efficient algorithm than *GTDS* that relied heavily on iteration and proved to be equally accurate. NIST was beginning to receive orbital elements for the GOES satellites at longer intervals than before (sometimes more than 30 d elapsed between sets of elements), so *GOESTRAK* was designed to predict the satellite’s position out to 50 d from the element’s epoch. Even with 50 d old elements, the service could meet its ±100 µs timing specification. Details of the orbital prediction algorithms were later published by NPLI [42].

*GOESTRAK* was originally used in the same fashion as *GTDS*. Orbital elements were entered into the software, and an ephemeris file was generated and uploaded to the TCGs at Wallops Island via a telephone connection. This procedure resulted in some occasional problems with data errors due to telephone line noise. In October 1999 a computer running a modified version of *GOESTRAK* was installed at Wallops Island. This computer was interfaced directly to the TCGs, and controlled from Boulder. This method proved to be extremely reliable during the final five years of the service.

## 11. GOES Applications

GOES time code receivers were once widely found and heavily relied upon at airports, electric power companies, scientific laboratories, observatories, and military installations. The NASDAQ stock exchange once relied on GOES as their master clock for stock market transactions, and the Smithsonian National Air and Space Museum in Washington, DC once had a small exhibit featuring a GOES time code receiver. When the Internet age began, GOES receivers became a source for Internet time synchronization [43], with software drivers written that allowed them to be used as reference clocks for Network Time Protocol (NTP) servers.

The largest users of the time code were probably the Federal Aviation Administration (FAA) and the electric power industry in both the United States and Canada. The FAA operates a system called Mode S, a secondary surveillance radar system that it uses to determine the position of aircraft moving in the airspace over the United States [44]. This system used GOES clocks for time stamping and sensor synchronization. Two GOES receivers, one for the east satellite and one for the west, were located at 147 United States airports, a total of 294 receivers. Additional receivers operated by the FAA brought the total number to well over 400. These units were replaced with GPS receivers in 2002 and 2003. The electric power industry once used thousands of GOES receivers to meet their synchronization requirements along the power grid, which was necessary to transfer power to the areas where it was needed most, and to quickly locate faults. However, the necessary level of synchronization for some applications began to approach 1 µs during the 1990s, a requirement that GPS could meet, but GOES could not [45].

The GOES effort also indirectly benefited other NIST time and frequency services. The technology developed for the GOES TCGs was later used in the original Automated Computer Time Service (ACTS) designed by NIST to synchronize computer clocks [46]. Perhaps more importantly, the experience gained by developing GOES time code receivers [35–37] no doubt played a role in the development of the first GPS common-view timing receiver in 1981 [47], which is still used today by NIST and other national laboratories for international time comparisons.

## 12. GOES Operations and NBS/NIST Cooperation With NOAA

After its initial development, the GOES time code service was efficiently operated, with minimal cost to NBS/NIST. In the latter years of the service, the oscillator(s) and GPS receiver(s) used as timing references were owned and maintained by NOAA. Six TCGs were built by NBS in the 1970s, and they proved to be exceptionally reliable, with three units still functioning at the end of 2004. The TCGs were controlled by the NIST staff from Boulder, requiring less than two hours of labor per week on average. NOAA personnel at the CDA were extremely helpful throughout the entire GOES time code era, replacing parts and helping solve problems whenever necessary. As a result, only three visits to the CDA were made by the NIST staff during the last nine years of the service (1996–2004).

NBS and NOAA signed three MOAs to provide the time code service. The first, in 1977, was for a 5 year period. This was renewed for five additional years in 1982, and for 10 additional years in 1987 [32]. When the final MOA expired in 1997, it was known that the service would not be continued indefinitely due to the declining number of customers. Therefore, the formal agreement was not renewed, but NIST and NOAA continued to provide the service on a “handshake” agreement until the January 1, 2005 termination date.

## 13. The Final Years of the GOES Service

When it became clear in the mid-1990s that most customers who needed high accuracy time were switching to GPS; the receiver manufacturers dropped their GOES products from their catalogs. In a short span of a year or two, all GOES time code receivers disappeared from the marketplace. However, there were some GOES customers who were perfectly happy with their equipment’s performance, and saw no reason to upgrade. To support these customers, one manufacturer actually released a product where a GPS receiver and antenna was contained inside a device that replaced the existing GOES antenna. The signals were converted to GOES format so that the existing GOES receiver could continue to be used.

NIST corresponded with many GOES customers throughout the final years of the service, keeping close tabs on their numbers, and helping many of them transition to new systems. Originally, a turn-off date of 2000 was considered, this was later moved to 2003. However, no formal announcement was made until 2002, when it became known that the FAA had procured over 400 GPS receivers to replace their existing GOES time code receivers. It was believed that the FAA’s decision to switch to GPS reduced the number of remaining GOES customers by more than half. As a result, a turn-off date of January 1, 2005 was proposed to NOAA in October 2002, and formally announced via press releases and the web sites of both agencies in early 2003, giving the remaining customers about 2 years to replace their receivers.

Even if a large user base had existed, it would have been necessary for NIST and NOAA to invest resources into the time code service to keep it going. The service had operated on a shoestring budget for many years and its age had begun to show. During the final 8 or 9 months of the service, the GOES/West time code was often unavailable due to problems with NOAA hardware at Wallops Island, but the still reliable GOES/East signal seemed to satisfy the few remaining customers, since it covered most of the United States (Fig. 7). The NIST TCGs and related hardware did an admirable job, lasting much longer than originally expected. However, the TCGs would have needed to be redesigned had the service continued, since repair parts were no longer available.

It was initially believed that NOAA might continue a reduced accuracy time code service without NIST involvement, since the time code was perhaps still used by NOAA for internal purposes. However, NOAA announced in May 2004 that the time code would be entirely removed from the broadcasts at the end of the year, and the time code service ended on January 1, 2005. In the week following the end of service, NIST heard from a number of customers who were still operating a total of more than 100 GOES time code receivers that were now obsolete. These customers included several utility companies located in the United States (one with 45 receivers), and several observatories located in North and South America. Most remaining customers had known about the end of the service, and after brief consultations with NIST were able to transition to new systems within a few days. However, one company had yet to complete their GPS procurement and asked for special assistance. As a result, NOAA temporarily broadcast a low accuracy time code from the satellites from January 13th until April 21st, allowing that company’s GOES receivers to resume operation until the transition to GPS was completed.

## 14. Summary

The story of the GOES time code service provides an excellent example of how NIST research can benefit the private sector and the American public. The direct offspring of satellite research conducted at NBS during the 1960s and 1970s, the GOES time code service went on to live a long and productive life. It made official United States time, accurate to 100 µs or better, readily available to the American public for three decades before being replaced by more modern technology.

## Footnotes

1 Certain commercial equipment, instruments, or materials are identified in this paper to foster understanding. Such identification does not imply recommendation or endorsement by NIST, nor does it imply that the materials or equipment identified are necessarily the best available for the purpose.
